# Cognitive reserve and the risk of postoperative neurocognitive disorders in older age

**DOI:** 10.3389/fnagi.2023.1327388

**Published:** 2024-02-05

**Authors:** Friedrich Borchers, Miriam Rumpel, Jochen Laubrock, Claudia Spies, Petra Kozma, Arjen Slooter, Simone J. T. van Montfort, Sophie K. Piper, Janine Wiebach, Georg Winterer, Tobias Pischon, Insa Feinkohl

**Affiliations:** ^1^Department of Anesthesiology and Intensive Care Medicine (CCM, CVK), Humboldt-Universität zu Berlin, Charité – Universitätsmedizin Berlin, Corporate Member of Freie Universität Berlin, Humboldt-Universität zu Berlin, Berlin, Germany; ^2^Department of Psychology, University of Potsdam, Potsdam, Germany; ^3^2nd Department of Internal Medicine and Nephrological Center, University of Pécs Medical School, Pécs, Hungary; ^4^Department of Intensive Care Medicine and Brain Center, University Medical Center Utrecht (UMC), Utrecht University, Utrecht, Netherlands; ^5^Institute of Medical Informatics, Charité – Universitätsmedizin Berlin, Corporate Member of Freie Universität Berlin, Humboldt-Universität zu Berlin, Berlin, Germany; ^6^Institute of Biometry and Clinical Epidemiology, Charité – Universitätsmedizin Berlin, Corporate Member of Freie Universität Berlin, Humboldt-Universität zu Berlin, Berlin, Germany; ^7^Pharmaimage Biomarker Solutions Inc., Cambridge, MA, United States; ^8^PI Health Solutions GmbH, Berlin, Germany; ^9^Molecular Epidemiology Research Group, Max-Delbrück-Center for Molecular Medicine in the Helmholtz Association (MDC), Berlin, Germany; ^10^Core Facility Biobank, Berlin Institute of Health at Charité – Universitätsmedizin Berlin, Corporate Member of Freie Universität Berlin, Humboldt-Universität zu Berlin, Berlin, Germany; ^11^Biobank Technology Platform, Max-Delbrück-Center for Molecular Medicine in the Helmholtz Association (MDC), Berlin, Germany; ^12^Charité – Universitätsmedizin Berlin, Corporate Member of Freie Universität Berlin, Humboldt-Universität zu Berlin, Berlin, Germany; ^13^Medical Biometry and Epidemiology Group, Witten/Herdecke University, Witten, Germany

**Keywords:** cognitive reserve, postoperative cognitive dysfunction, postoperative neurocognitive disorders, postoperative delirium, elective surgery, geriatric assessment

## Abstract

**Background:**

Postoperative delirium (POD) and postoperative cognitive dysfunction (POCD) are postoperative neurocognitive disorders (PNDs) that frequently occur in the aftermath of a surgical intervention. Cognitive reserve (CR) is a concept posited to explain why cognitive health varies between individuals. On this qualitative understanding of cognitive health, factors like IQ, education level, and occupational complexity can affect the impact of neuropathological processes on cognitive outcomes.

**Methods:**

We investigated the association between CR and POD and CR and POCD on data from 713 patients aged≥65 years with elective surgery. Peak pre-morbid IQ was estimated from vocabulary. Occupational complexity was coded according to the Dictionary of Occupational Titles (DOT). Education level was classed according to the International Standard Classification of Education (ISCED). These three factors were used as proxies of CR. In a series of regression models, age, sex, depression, site of surgery, and several lifestyle and vascular factors were controlled for.

**Results:**

Patients with a higher IQ had lower odds of developing POD. We found no significant association between the other two CR markers with POD. None of the CR markers was associated with POCD.

**Conclusion:**

The significant association of a higher IQ with lower POD risk allows for the stratification of elderly surgical patients by risk. This knowledge can aid the prevention and/or early detection of POD. Further research should attempt to determine the lack of associations of CR markers with POCD in our study.

## 1 Introduction

Cognitive change after anesthesia and surgery can occur in any age group but is most common among geriatric patients (Moller et al., 1998). A postoperative decline in cognitive function is as-associated with decreased quality of life, increased length of hospital stay, increased care dependency, and increased mortality (Abelha et al., 2013; Ying Tan and Amokao, 2013; Kastaun et al., 2016). These negative treatment outcomes increase the costs of health and/or social care (Steinmetz et al., 2009; Rudolph and Marcantonio, 2011).

In 2019, more than 17.23 million operations were carried out in German hospitals, of which 20 percent (3.6 million) were carried out on patients aged 60 or above (Statistisches Bundesamt, 2020). As the population of Europe ages, strategies for the prevention, detection, and treatment of postoperative neurocognitive disorders (PNDs) will take on more importance. Preoperative geriatric assessment should include cognitive assessment and be targeted at risk reduction for the individual patient. Understanding of PND risk factors may serve to recommend structured preoperative assessment. The known risk factors of PNDs include advanced age, comorbid cerebral, cardiac, and vascular diseases, and pre-existing cognitive problems, as well as further intra- and postoperative factors (Levin, 2007; Whitlock et al., 2011; Rundshagen, 2014; Aldecoa et al., 2017). Knowledge of these risk factors should be applied to patient risk stratification, informed clinical decision-making, and targeted patient care in the perioperative period (Feinkohl, 2022). These strategies could reduce the number of cases of postoperative delirium (POD) and postoperative cognitive dysfunction (POCD), improve patient outcomes, and reduce expenditures for health and social care providers (Zhang et al., 2017). The incidence of PNDs depends on the composition of risk factors that are specific for different surgical cohorts (e.g., vascular changes, metabolic factors, inflammation after surgery, chronic pain, and others): in cardiac bypass surgery the incidence of POD ranges from 37 to 52% compared to 5–51% in abdominal surgery (Rudolph and Marcantonio, 2011). In cohorts with total knee arthroplasty, most studies report a POD incidence of 10–15% (Kitsis et al., 2022). POCD was most often investigated in subjects with cardiac surgery, followed by vascular surgery, orthopedic surgery, and lastly abdominal surgery. Reported incidences are strongly related to the composition of the cognitive test battery, the time point of testing, and diagnostic rules (Borchers et al., 2021).

Cognitive health varies between individuals in all stages of life and affects an individual’s resilience to damage to the brain due to age, injury, or disease (Montine et al., 2019). Pre-morbid intelligence quotient (IQ), education level, and occupation level are factors thought to be protective against damage to the brain (Ko et al., 2022). The concept that brain function can determine an individual’s resilience to cognitive damage is known as cognitive reserve (CR) (Stern et al., 2019). In this manuscript, we focus on the association of cognitive reserve factors with POD, “an acute disturbance in both attention and awareness” occurring in the immediate aftermath of an operation (Feinkohl et al., 2017; Sawamura, 2017), and POCD, a relevant change in cognitive function compared to the preoperative period diagnosed earliest 30 days up to 1 year after surgery (Evered et al., 2018). The association between CR and POD/POCD has not been adequately studied. A comprehensive overview of the current state of research and literature addressing the relationship between CR and POD and POCD was given in a 2022 narrative review (Feinkohl, 2022). The review reported conflicting results for the association of education level with POD. While education level was the most extensively explored CR variable, only two studies assessed pre-morbid IQ as a risk factor of POD, again with contradictory results (Schmitt et al., 2015; Hill et al., 2018). The Successful Aging After GEneral Surgery study (SAGES) was the only study that included occupation data in the context of POD (Saczynski et al., 2014). However, neither occupational complexity nor managerial demands were found to be associated with POD (*Ibid.*). Regarding POCD, the association with the highest attained education level has been well explored, and the evidence here is more cohesive (Feinkohl, 2022). In a 2017 meta-analysis of 15 studies covering four continents and follow-up until 6 months after surgery, each additional year in education was associated with a 10% lower POCD risk (Feinkohl et al., 2017). Two studies assessed pre-morbid IQ as a risk factor for POCD, with neither finding an association (Medi et al., 2013; Scott et al., 2017). A single study to date has examined patient occupation in the context of POCD, finding no association. However, the statistical power of this study was limited due to its small sample size (Relander et al., 2020).

Here, we used a cohort of older surgical patients to assess whether a higher level of CR – understood in terms of having a higher IQ, a higher level of education, or a higher level of occupational complexity – is associated with a lower risk of developing POD and POCD. By controlling for potential confounders, we determined the independence of the expected associations. We also determined the contribution of potential mediators including vascular risk factors. We chose to exclude patients screened positively for pre-existing major neurocognitive disorders. We did so in view to capture the surgery-induced cognitive decline/POD that occurs in the general older population, rather than in the specific subpopulation with cognitive impairment as has been introduced by the International Study of Post-Operative Cognitive Dysfunction (ISPOCD) working group, one of the pioneer multicenter studies in the area of POCD research in 1996 (Moller et al., 1998).

## 2 Methods

### 2.1 Study aim and design

We used data from the Biomarker Development for Postoperative Cognitive Impairment in the Elderly (BioCog) study (Winterer et al., 2018). The study aimed to establish valid biomarkers for risk and clinical outcome prediction of POD/POCD. The study was approved by the local medical ethics committees of the study centers in Berlin, Germany (EA2/092/14), and Utrecht, Netherlands (14–469) and registered at clinicaltrials.gov under NCT02265263.

### 2.2 Patient recruitment, data collection, and clinical assessments

We included patients aged ≥65 scheduled for any elective surgical procedure with an expected duration of at least 60 min. The site of surgery was classified as a binary variable differentiating intrathoracic, intraabdominal, and pelvic surgery from peripheral surgery. We excluded patients with intracranial surgery, with a Mini-Mental Status Examination (MMSE) score of ≤23 points, severe hearing or visual impairment, and/or neuropsychiatric disease interfering with neuropsychological testing.

Clinical, sociodemographic, and lifestyle data, and cognitive and laboratory data relevant to our secondary analysis were collected in structured interviews, clinical examination, questionnaires, and patient files before and 3–6 months after surgery. Anesthesia was not standardized and performed based on the clinical choice of the respective anesthesiologist.

### 2.3 Pre-morbid IQ

Vocabulary skills were used for approximating pre-morbid IQ, as they are resilient to age-related cognitive decline. The vocabulary skills of German patients were tested using the Mehrfachwortschatztest A (MWT-A) (Lehrl et al., 1991), the German version of the Mill-Hill Vocabulary Scale (MHVS) (Lehrl et al., 1995). For Dutch patients, the Dutch Adult Reading Test (DART) was used (Schmand et al., 1991). DART and MWT-A scores were converted to intelligence quotient (IQ) ranks based on published norms (Schmand et al., 1991; Fischer, 2001). Further details are accessible in the Supplementary material.

### 2.4 Occupational complexity

Last occupation prior to retirement (or current occupation) was self-reported by patients. The last occupation was recorded in German at the Berlin sites and in English in Utrecht. German occupation data were translated into English by a bilingual native speaker. The Dictionary of Occupational Titles (DOT) (National Academy of Sciences, Committee on Occupational Classification and Analysis, 1981) was then used to assess the occupational complexity of patients’ last occupation in terms of three factors: “data,” “people,” and “things.” A detailed description is provided in the Supplementary material.

### 2.5 Education level

Full patient educational background was classified according to the International Standard Classification of Education (ISCED). In Berlin, ISCED 1997 (International Standard Classification of Education ISCED, 1997) was used, which assigns categories ranging from 0 to 6 to each individual: 0, ‘pre-primary level of education’; 1, ‘primary’; 2, ‘lower secondary’; 3, ‘upper secondary’; 4, ‘post-secondary non-tertiary’; 5, ‘first stage tertiary’; 6, ‘second stage tertiary’. For the purpose of the current analysis, participants were grouped into ‘ISCED 1/2’, ‘ISCED 3/4’, and ‘ISCED 5/6’. At the Dutch study center, the ISCED 2011 classification system was used (International Standard Classification of Education ISCED, 2011) and converted to the ISCED 1997 based on the ISCED 1997–2011 conversion table presented in Table S3 in the Supplementary material. None of the patients had ISCED 0.

### 2.6 Psychological comorbidity, lifestyle, and vascular health

Depressive symptoms in patients were assessed using the 15-item Geriatric Depression Scale (GDS) (Gauggel and Birkner, 1999). A GDS score of 0–4 points was classified as “normal,” and 5–9 points as “mild or moderate depression,” while a score of 10 or more was considered to indicate “severe depression.”

Patients were attributed past or present smoking status, and nutritional status was assessed with the Body Mass Index (BMI) and Short Form of the Mini Nutritional Assessment (MNA-SF) (MNA-International Group et al., 2009). We built models to control for smoking, obesity, diabetes, and hypertension as lifestyle risk factors and comorbid health conditions known to be inversely correlated with level of education, occupation, and IQ (Cavelaars et al., 2000; Smith, 2007; Der et al., 2009). These factors are detrimental to overall health – including brain health (Cox et al., 2019) – which could explain, in part, why those with a higher level of CR experience better postoperative cognitive outcomes. Furthermore, we included laboratory markers of vascular health [High-Density-Lipoprotein- Cholesterin (HDL), Triglycerides (TG), and glycated hemoglobin (HbA1c)] in our analysis models (Saudek and Brick, 2009; Impact of High Cholesterol on Vascular Health, n.d.). These were taken on the day of surgery when patients were generally fasting.

### 2.7 Assessment of POD

Screening for POD started in the recovery room and was repeated twice daily at 8:00 am and 7:00 pm (+/− 1 h), except for weekend days, for up to 7 days after surgery, or until discharge from hospital, if this occurred first. The screening was conducted by a clinical research team trained and supervised by psychiatrists and delirium experts, independently of routine hospital procedures. Assessments included simultaneous scoring of sedation level and pain according to evidence-based and consensus-based guidelines on postoperative delirium recommendations (Aldecoa et al., 2017). POD was defined according to the 5th edition of the Diagnostic and Statistical Manual of Mental Disorders (DSM-5) criteria. Patients were considered delirious if they

- scored ≥2 cumulative points on the Nursing Delirium Screening Scale (Nu-DESC) (Gaudreau et al., 2005; Lütz et al., 2008) and/or
- had a positive Confusion Assessment Method (CAM) score and/or
- had a positive score for the CAM for the Intensive Care Unit (CAM-ICU) (Hestermann et al., 2009) and/or
- showed signs of delirium (e.g., confusion, agitation, drowsiness, disorientation, prescription of antipsychotics) in their treatment chart.

POD assessment methods are detailed in the Supplementary material.

### 2.8 Assessment of POCD

Patients underwent neurocognitive testing at three time points: before surgery, during the week after surgery, and 3–6 months after surgery. Tests consisted of a comprehensive screen-based neuropsychological test battery (CANTAB®, Cambridge Cognition Ltd., Cambridge, UK), the trail-making test, and the grooved pegboard test. Trained study assistants performed testing in accordance with a standard operating procedure (SOP) developed by two neuropsychologists. Two independent assessors performed data plausibility checks. Incomplete test values were imputed: for data missing due to a lack of concentration or understanding of the test, the worst performance data for the entire patient group were used as a substitute (11 cases – 1.5%); for data missing at random, e.g., due to technical errors, random forest imputation was applied (missForest package for R Statistical Software) (37 cases – 5.2%). No imputation was applied for complete neuropsychological testing missing at a given follow-up.

A non-operated comparison group was recruited from outpatient clinics, primary care, nursing homes, and during public talks. The 114 subjects in the non-operated comparison group had identical inclusion/exclusion criteria with the intervention group except for surgery, and completed neuropsychological testing at baseline, 1 week, and 3 months after baseline. Subject demographics of the non-operated comparison group are provided in Supplementary Table S4. Data on the stability of the neuropsychological test performance of this cohort serving as normative control have been published (Feinkohl et al., 2020). The neurocognitive test battery and diagnostic rule for POCD are detailed in the Supplementary material.

### 2.9 Statistical analyses

We reorganized the database in SPSS (IBM, version 27) and used R Statistical Software (R Core Team, version 4.1.1) for data analysis. The level of statistical significance was set at *p* < 0.05 without adjustment for multiple testing. All value of *p*s constitute exploratory analysis.

### 2.10 Treatment of missing and unsuitable data

Patients with missing data on the key variables of POD, POCD, IQ, last occupation, and/or education level were excluded from the analysis sample.

**Figure 1:**
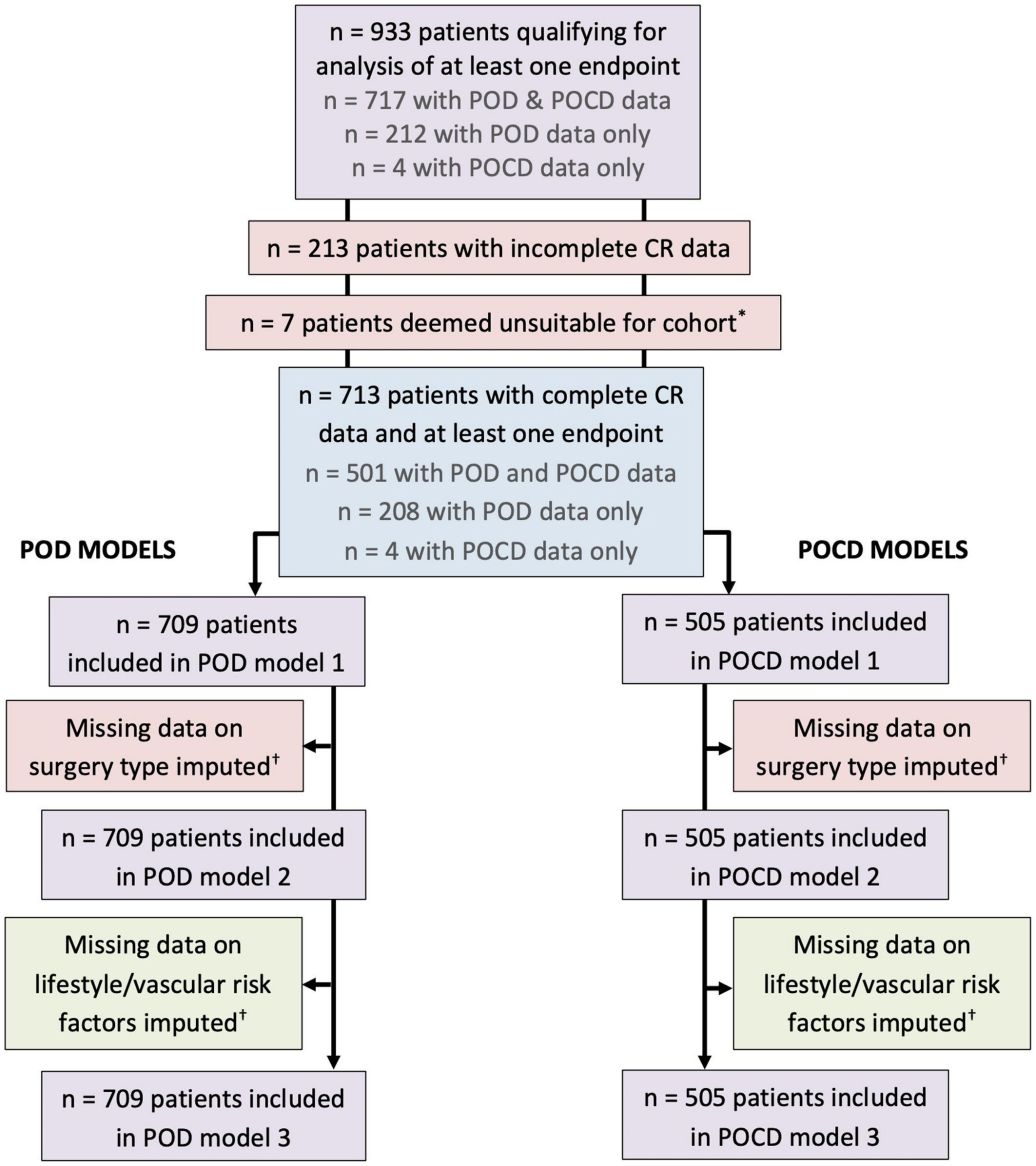
Inclusion chart. *Three patients underwent operations to fit a shunt to treat hydrocephalus, two patients had a stimulation device implanted to treat an essential tremor, and two patients had surgeries on brain tumors. These were not included in the analysis sample. ^†^Missing data on site of surgery (*n* = 20), GDS (*n* = 44), current smoking status (*n* = 16), hypertension (*n* = 11), HDL (*n* = 131), TG (*n* = 132), HbA1c (*n* = 155). For missing categorical data, the most common category was imputed, i.e., “peripheral” for site of surgery, “no” for current smoking, and “yes” for hypertension. For missing continuous data the median was imputed.

For missing of categorical data [site of surgery (*n* = 20), current smoking status (*n* = 16), hypertension (*n* = 11)], the most common category was imputed, i.e., “peripheral” for site of surgery, “no” for current smoking, and “yes” for hypertension. For absences of continuous data [GDS (*n* = 44), HDL (*n* = 131), TG (*n* = 132), HbA1c (*n* = 155)] the median value was imputed (Figure 1).

Extreme values of the CR marker “occupational complexity: people” (range 0–8) were rather due to a class imbalance issue and therefore included in the analysis. Of the control variables, extreme outliers were found in the lifestyle and vascular health variables BMI (range 14.73–46.77 kg/m^2^), HDL (range 0.10–2.93 mmol/L), TG (range 0.31–17.10 mmol/L), and HbA1c (range 15.30–88.00 mmol/mol). Extreme low values were replaced with the 10th percentile value and extreme high values with the 90th percentile value. The lower outer fence was defined as Q1 – (3IQR) and the upper outer fence as Q3 + (3IQR), where Q1 and Q3 were the lower and upper quartiles, respectively, and IQR the interquartile range.

### 2.11 Preliminary analyses

Descriptive statistics are given as mean with standard deviation (SD), median with limits of the interquartile range (IQR), or absolute numbers and relative frequencies, depending on scale.

Analyses of variance (ANOVAs) were used to compare pre-morbid IQ across education groups and between male and female patients. Kruskal-Wallis Tests were used to compare occupational complexity across education groups and between male and female patients. A χ^2^ test was used to examine the association of education with sex.

The Spearman correlation coefficient was calculated to examine the association between occupational complexity and pre-morbid IQ.

### 2.12 Logistic regression models

For the main analyses, multivariate logistic regression models were used to estimate odds ratios (OR) with 95% confidence intervals (CI) for the occurrence of POD and POCD, respectively. We entered all independent variables (IQ, Education level, and Occupational complexity) together in each model. Occupational complexity was used as a continuous variable. Each analysis involved three steps: In model 1, age and sex were controlled for. In model 2, depression score and site of surgery were additionally controlled for. In model 3, lifestyle and vascular factors (current smoking, BMI, diabetes, hypertension, HDL, TG, and HbA1c) were additionally controlled for to determine the potential role of these factors as mediators to the expected associations.

## 3 Results

The analysis sample contained 713 patients aged 65–90 years (median 72 years) with complete data on IQ, education, and occupation. Of these patients, 709 had POD data, while 505 had POCD data (Figure 1). Demographic, cognitive, and medical characteristics of these patients are summarized in Table 1. Patients were of a relatively high education level and IQ. Occupational complexity scores varied for “data” (IQR 3–5), “people” (IQR 2–3), and “things” (IQR 0–6).

**Table 1:** Patient characteristics.

Total sample *n* = 713		Median	IQR
Age (years)		72.0	68–75
MMSE score (points)		29.0	28–30
GDS score (points)		1.0	0–3
BMI (kg/m^2^)		26.6	24.0–29.2
HDL (mmol/L)		1.3	1.1–1.6
TG (mmol/L)		1.5	1.2–1.8
HbA1c (mmol/mol)		35.0	32.8–37.2
IQ		111	102–123
Occupational complexity	Data	4	3–5
	People	2	2–3
	Things	3	0–6
		Absolute number	Relative frequency (%)
Female		305	43
Site of surgery	Intrathoracic, -abdominal, -pelvic	323	45
	Peripheral	390	55
Current smoker		67	9
Hypertension		455	64
Diabetes		151	21
Education level	ISCED 1/2	109	15
	ISCED 3/4	296	42
	ISCED 5/6	308	43
POD (*n* = 709)		146	21
POCD (*n* = 505)		49	10
Died before 3 months follow-up		24	3

MMSE, Mini-Mental-State-Exam; GDS, Geriatric Depression Scale; BMI, Body Mass Index; TG, triglycerides; HDL, High-Density-Lipoprotein-Cholesterin; HbA1c, glycated hemoglobin; IQ, intelligence quotient; ISCED, International Standard Classification of Education; POD, Postoperative delirium; POCD, postoperative cognitive dysfunction. No listwise deletion of missing data on surgical factors, lifestyle factors, and comorbidities was undertaken. Data for the site of surgery was missing for 20 patients; GDS data was missing for 44 patients; data on current smoking was missing for 16 patients; data on hypertension was missing for 11 patients. Initially, diabetes data was missing in the database for 10 patients from Berlin, however, this data was successfully extracted from patient files and amended in the database. BMI data was complete for all patients. Concerning blood values, 131 patients were missing HDL data, 132 were missing TG data, and 155 were missing HbA1c data. For missing continuous variables, the median was imputed; for missing categoric data, the most common category was imputed, i.e., “peripheral” for the site of surgery, “no” for current smoking, and “yes” for hypertension.

Depression scores were relatively low (median 1.0 of maximum 15 points). Only 40 patients (5.6%) scored higher than four points on GDS indicating mild to severe depressive symptoms.

The majority of patients in the sample were overweight (median BMI of 26.6 kg/m^2^). Nine percent of patients were current smokers, and the prevalences of hypertension and diabetes were 62 and 21%, respectively.

Median patient TG levels were 1.5 mmol/L, median HDL levels 1.3 mmol/L, and median HbA1c levels 35.0 mmol/mol.

Male and female patients did not differ significantly in age or IQ. Education level was significantly associated with sex, with male patients having higher levels of education. Jobs held by men were more complex with regard to “data” and “people” but did not differ in occupational complexity with “things.” Gender differences are shown in more detail in the Supplementary Table S2.

As shown in Table 2 patient education level was significantly associated with pre-morbid IQ (*p* < 0.001). Patients in the middle education level (ISCED 3/4) had higher IQs than those in the lower education level (ISCED 1/2), while those in the highest level (ISCED 5/6) had higher IQs again. All three occupational complexity factors were significantly associated with education level (*p* < 0.001). For complexity with “data” and “people,” patients in the lowest education level had the lowest complexity scores, and patients in the highest education level the highest. For occupational complexity related to “things,” the pattern was different: patients in the middle education level (ISCED 3/4) had the highest occupational complexity ratings, followed by patients in the lowest education level (ISCED 1/2), while those in the highest education level (ISCED 5/6) had the lowest complexity ratings related to “things” of the three groups. Correlations between IQ and occupational complexity were low for both “data” complexity and “people” complexity (rho = 0.26, *p* < 0.001 and rho = 0.27, *p* < 0.001 respectively). There was insufficient evidence of a correlation between IQ and occupational complexity with respect to “things” (rho = −0.07, *p* = 0.052). Scatterplots for the correlation of occupational complexity with IQ are shown in the Supplementary Figures S1–S3.

**Table 2:** Associations between IQ, education level, and occupational complexity.

	**Education level**				
	**ISCED 1/2**	**ISCED 3/4**	**ISCED 5/6**	***F*-value**	**Value of *p***
**IQ** **[Mean ± SD]**	100.9 ± 13.8	108.6 ± 14.2	118.1 ± 11.9	81.03	<0.001
**Occupational complexity** **[Median (IQR)]**				***Χ***^**2**^**(2, *n* = 713)**	
**Data**	3 (1–4)	3.5 (3–5)	5 (4–5)	172.61	<0.001
**People**	2 (0–2)	2 (2–2)	2 (2–6)	125.77	<0.001
**Things**	3 (0–5)	4 (0–6)	0 (0–6)	16.88	<0.001
	**IQ**			**Rho**	**Value of *p***
**Occupational complexity**					
**Data**				0.26	<0.001
**People**				0.27	<0.001
**Things**				−0.07	0.052

Analyses of variance (ANOVAs) were used for comparison of pre-morbid IQ across education groups (*F*- and value of *p*s provided). Kruskall–Wallis Tests were used for the comparison of occupational complexity variables across education groups (*χ*^2^ and value of *p* provided). The Spearman correlation coefficient was calculated to test for the correlation of occupational complexity variables with IQ (rho and value of *p* provided).

The results from the analysis of the association between CR and postoperative NCDs are shown in Tables 3, 4. The only significant association found was between IQ and POD, such that patients with a higher IQ had lower odds of developing POD. In model 1, adjusting for age and sex, for every one unit increase in IQ, the odds of having POD decreased by 3% (Odds Ratio 0.97, 95% CI 0.95–0.98, *p* < 0.001). We standardized this measure: for every one SD increase in IQ, the odds of having POD decreased by 37% (OR 0.63, 95% CI 0.51–0.78, p < 0.001). In models 2 and 3, the effect remained significant and the effect size was stable. CR was not associated with POCD (Table 4). We did not find differences in CR markers between patients with peripheral versus intraabdominal/−thoracic/−pelvic site of surgery (Supplementary Table S5). CR markers were not associated with mortality (data provided in the Supplementary material).

**Table 3:** POD models 1–3 for IQ, occupational complexity, and education.

*n* = 709	Model 1: adjusted for age and sex	
	OR (95% CI)	Value of *p*
IQ	0.97 (0.95, 0.98)	<0.001
Occupational complexity		
Data	1.09 (0.95, 1.25)	0.216
People	0.96 (0.87, 1.07)	0.483
Things	1.02 (0.95, 1.11)	0.558
Education level		
ISCED 3/4*	1.00 (0.57, 1.80)	0.996
ISCED 5/6*	1.80 (0.92, 3.59)	0.091
	Model 2: additionally adjusted for depression and site of surgery	
	OR (95% CI)	Value of *p*
IQ	0.97 (0.96, 0.99)	<0.001
Occupational complexity		
Data	1.10 (0.96, 1.27)	0.172
People	0.95 (0.85, 1.06)	0.363
Things	1.04 (0.96, 1.13)	0.329
Education level		
ISCED 3/4*	0.97 (0.54, 1.78)	0.908
ISCED 5/6*	1.84 (0.92, 3.76)	0.089
	Model 3: additionally adjusted for lifestyle and vascular risk factors	
	OR (95% CI)	Value of *p*
IQ	0.97 (0.96, 0.99)	0.003
Occupational complexity		
Data	1.15 (0.98, 1.35)	0.097
People	0.96 (0.85, 1.08)	0.499
Things	1.06 (0.97, 1.17)	0.192
Education level		
ISCED 3/4*	1.12 (0.57, 2.29)	0.732
ISCED 5/6*	1.68 (0.76, 3.87)	0.211

IQ, occupational complexity, and education were entered together in each regression model. *Compared to ISCED 1/2.Nagelkerke *R*^2^ in Model 1: 0.10; Model 2: 0.17; Model 3: 0.50.

**Table 4:** POCD models 1–3 for IQ, occupational complexity, and education.

*n* = 505	Model 1: adjusted for age and sex	
	OR (95% CI)	Value of *p*
IQ	1.00 (0.98, 1.02)	0.995
Occupational complexity		
Data	1.04 (0.84, 1.30)	0.728
People	0.93 (0.77, 1.10)	0.394
Things	1.08 (0.96, 1.22)	0.214
Education level		
ISCED 3/4*	1.23 (0.50, 3.38)	0.662
ISCED 5/6*	1.39 (0.47, 4.46)	0.568
	Model 2: additionally adjusted for depression and site of surgery	
	OR (95% CI)	value of p
IQ	1.00 (0.98, 1.03)	0.864
Occupational complexity		
Data	1.05 (0.85, 1.31)	0.661
People	0.92 (0.77, 1.09)	0.341
Things	1.08 (0.96, 1.23)	0.219
Education level		
ISCED 3/4*	1.25 (0.50, 3.43)	0.646
ISCED 5/6*	1.43 (0.48, 4.63)	0.531
	Model 3: additionally adjusted for lifestyle and vascular risk factors	
	OR (95% CI)	value of p
IQ	1.00 (0.97, 1.03)	0.814
Occupational complexity		
Data	1.08 (0.84, 1.42)	0.554
People	0.99 (0.81, 1.19)	0.888
Things	1.05 (0.91, 1.22)	0.499
Education level		
ISCED 3/4*	1.36 (0.45, 4.70)	0.603
ISCED 5/6*	1.62 (0.44, 6.88)	0.490

IQ, occupational complexity, and education were entered together in each regression model. *Compared to ISCED 1/2. Nagelkerke *R*^2^ in Model 1: 0.09; Model 2: 0.10; Model 3: 0.42.

## 4 Discussion

We found that higher pre-morbid IQ was associated with a lower odds of developing POD. When including lifestyle and vascular risk factors in a logistic regression model the lower odds of POD in higher IQ individuals were not due to the fact that these individuals were physically healthier in terms of their BMI, smoking habits, comorbid diabetes and hypertension, and vascular health. This indicates that lifestyle and vascular risk factors are not mediators between IQ and POD. Contrary to our expectation, occupational complexity of “data,” “people,” and “things” or education level were not associated with POD. Further, no association between any of the CR markers and POCD was found.

The results of this analysis are in line with those of the SAGES study, showing a negative association between IQ and POD incidence, and no association between occupation and POD incidence (Saczynski et al., 2014). Using the Wechsler Test of Adult Reading (WTAR) as a basis for understanding IQ, Saczynski et al. found that an 0.5 SD increase in WTAR score was associated with a 38% reduction in POD risk (*Ibid.*) – a very similar finding to that of the present study. The authors of the paper concluded that “the [CR] markers that are important for [POD] may be different from those considered to be important for dementia” (*Ibid.*).

It was surprising that the present analysis did not find an association between either POD or POCD and education, the CR variable most commonly explored with respect to PNDs (Feinkohl, 2022). While the literature reports conflicting results on the association of education and POD including studies which, like in our analyses, found no association between POD and education (*Ibid.*), POCD is known to be strongly associated with education (Feinkohl et al., 2017). We may not have been able to replicate the association of education level with POCD as we used ISCED categories instead of a continuous variable, e.g., education years, in our analyses.

The strong association of POD risk with patient IQ, discerned through a vocabulary test (MWT-A, DART), represents an interesting contribution to the discussion of whether POD and POCD are to be understood as separate disorders or as different manifestations of the same underlying dysfunction (Devinney et al., 2018). On long-term follow-up of the SAGES cohort “delirium was associated with a 40% acceleration in the slope of cognitive decline out to 72 months following elective surgery” (Kunicki et al., 2023). The results of our analysis show that a lower IQ is a significant risk factor for a patient developing POD but has no bearing on their POCD risk. Patients with higher IQ may compensate for attention deficits better than patients with lower IQ, be affected by subthreshold delirious symptoms instead, and still be at risk of developing POCD. Higher cognitive reserve may therefore not be protective but a risk of unnoticed perioperative cognitive disturbance.

### 4.1 Limitations

The choice of the CR markers used was constrained by the data available. Other variables may have been useful additional markers to consider. Lifetime cognitive engagement would be especially interesting as a CR marker as this – potentially used in combination with occupational data – draws a more holistic picture of an individual’s cognitive history than occupation alone, which only represents part of an individual’s cognitive activities over the course of their life. The three reserve parameters may be converted into a composite to segment patients into groups of ‘low -’ and ‘high cognitive reserve’ (Feinkohl, 2022). Though of potential interest, we did not use data collected on patients’ alcohol consumption in our analysis due to concerns about the reliability of the self-reported measures (Egholm et al., 2018). Furthermore, the inclusion of patients independent of their pre-operative cognitive status may change the association of CR factors with perioperative cognitive reserve, as preoperative cognitive impairment has been described as an independent risk factor of PNDs (Culley et al., 2017). Precipitating factors, e.g., body-weight-adjusted anesthesia dosages and intraoperative sedation depth may be associated with PNDs, but the systematic review does not support this assumption (Wang et al., 2023). We are convinced that future investigations of modifiable precipitating factors require research designs that standardize anesthesia and postoperative treatment to control for the complexity of PND etiology in individualized patient care. We therefore chose to limit our analyses of data from this observational study to predisposing factors and site of surgery. With this approach, we focus on the association of CR factors with PNDs from an epidemiological perspective and variables detectable during preoperative risk assessment.

None of the three occupational complexity variables were associated with either POD or POCD. Longest-held occupation may have been a more fitting CR factor compared to last-held occupation, though in the SAGES study, longest-held occupation was not associated with occupational complexity and POD either (Saczynski et al., 2014).

Limited variance of ISCED as a categorical variable may have hindered finding an association between education level and POCD that was reported in a meta-analysis which found a significant difference in POCD risk between patients who had completed tertiary education and those who had completed only secondary education (Feinkohl et al., 2017).

Several more challenges are typical of studies on perioperative cognitive trajectories. The patient’s level of anxiety during cognitive testing on the day before surgery may have altered baseline cognitive performance. Dropout is common for organizational reasons, poor patient physical or mental health, and mortality. Furthermore, it is unsurprising that data across a number of variables were incomplete within a multi-site clinical study. In consequence, data imputation could also have impacted the results.

## 5 Conclusion

This study has identified a significant association between IQ and POD such that for every one SD increase in IQ, the odds of having POD decreased by 37%. No other CR variable was associated with either POD or POCD. More research on the association of CR and PNDs is needed to assess if high cognitive reserve reduces brain vulnerability in the perioperative period or rather increases the risk of perioperative disturbance remaining undetected in current screening strategies. Studies should apply composite CR measures with highly relevant variables, e.g., longest-held occupation and lifetime engagement in cognitive activities. POD assessment should allow for the detection of subthreshold (sub-syndromal) delirium.

## Data availability statement

The raw data supporting the conclusions of this article will be made available by the authors, without undue reservation.

## Ethics statement

The studies involving humans were approved by the medical ethics committee, Charité – Universitätsmedizin Berlin, corporate member of Freie Universität Berlin, Humboldt-Universität zu Berlin, Berlin, Germany and the medical ethics committee, Utrecht University, Utrecht, the Netherlands. The studies were conducted in accordance with the local legislation and institutional requirements. The participants provided their written informed consent to participate in this study.

## Author contributions

FB: Conceptualization, Data curation, Formal analysis, Investigation, Methodology, Writing – original draft. MR: Conceptualization, Data curation, Formal analysis, Investigation, Methodology, Writing – original draft. JL: Supervision, Writing – review & editing. CS: Funding acquisition, Resources, Supervision, Writing – review & editing. PK: Data curation, Writing – review & editing. AS: Investigation, Resources, Writing – review & editing. SM: Data curation, Investigation, Writing – review & editing. SP: Methodology, Supervision, Writing – review & editing. JW: Formal analysis, Methodology, Writing – review & editing. GW: Funding acquisition, Project administration, Resources, Supervision, Writing – review & editing. TP: Resources, Writing – review & editing. IF: Conceptualization, Data curation, Formal analysis, Methodology, Supervision, Writing – original draft.

## References

[ref1] Abelha F. J. Luís C. Veiga D. Parente D. Fernandes V. Santos P.. (2013). Outcome and quality of life in patients with postoperative delirium during an ICU stay following major surgery. Crit. Care 17:R257. doi: 10.1186/cc13084, PMID: 24168808 PMC4057091

[ref2] Aldecoa C. Bettelli G. Bilotta F. Sanders R. D. Audisio R. Borozdina A.. (2017). European Society of anaesthesiology evidence-based and consensus-based guideline on postoperative delirium. Eur. J. Anaesthesiol. 34, 192–214. doi: 10.1097/EJA.0000000000000594, PMID: 28187050

[ref4] Borchers F. Spies C. D. Feinkohl I. Brockhaus W. R. Kraft A. Kozma P.. (2021). Methodology of measuring postoperative cognitive dysfunction: a systematic review. Br. J. Anaesth. 126, 1119–1127. doi: 10.1016/j.bja.2021.01.035, PMID: 33820655

[ref5] Cavelaars A. E. Kunst A. E. Geurts J. J. Crialesi R. Grötvedt L. Helmert U.. (2000). Educational differences in smoking: international comparison. BMJ 320, 1102–1107. doi: 10.1136/bmj.320.7242.1102, PMID: 10775217 PMC27351

[ref6] Cox S. R. Lyall D. M. Ritchie S. J. Bastin M. E. Harris M. A. Buchanan C. R.. (2019). Associations between vascular risk factors and brain MRI indices in UK biobank. Eur. Heart J. 40, 2290–2300. doi: 10.1093/eurheartj/ehz100, PMID: 30854560 PMC6642726

[ref7] Culley D. J. Flaherty D. Fahey M. C. Rudolph J. L. Javedan H. Huang C. C.. (2017). Poor performance on a preoperative cognitive screening test predicts postoperative complications in older orthopedic surgical patients. Anesthesiology 127, 765–774. doi: 10.1097/ALN.0000000000001859, PMID: 28891828 PMC5657553

[ref8] Der G. Batty G. D. Deary I. J. (2009). The association between IQ in adolescence and a range of health outcomes at 40 in the 1979 US National Longitudinal Study of youth. Intelligence 37, 573–580. doi: 10.1016/j.intell.2008.12.002, PMID: 19907663 PMC2772900

[ref9] Devinney M. J. Mathew J. P. Berger M. (2018). Postoperative delirium and postoperative cognitive dysfunction: two sides of the same coin? Anesthesiology 129, 389–391. doi: 10.1097/ALN.0000000000002338, PMID: 29965817 PMC6092234

[ref10] Egholm J. W. Pedersen B. Møller A. M. Adami J. Juhl C. B. Tønnesen H.. (2018). Perioperative alcohol cessation intervention for postoperative complications. Cochrane Database Syst. Rev. 2018:CD008343. doi: 10.1002/14651858.CD008343.pub3, PMID: 30408162 PMC6517044

[ref11] Evered L. Silbert B. Knopman D. S. Scott D. A. DeKosky S. Rasmussen L. S.. (2018). Nomenclature consensus working group. Recommendations for the nomenclature of cognitive change associated with anaesthesia and Surgery-2018. Anesth. Analg. 127, 1189–1195. doi: 10.1213/ANE.0000000000003634, PMID: 30325748

[ref12] Feinkohl I. (2022). Post-operative cognitive impairment: a cognitive epidemiology perspective. J. Intelligence 10:18. doi: 10.3390/jintelligence10010018, PMID: 35324574 PMC8949407

[ref13] Feinkohl I. Borchers F. Burkhardt S. Krampe H. Kraft A. Speidel S.. (2020). Stability of neuropsychological test performance in older adults serving as normative controls for a study on postoperative cognitive dysfunction. BMC. Res. Notes 13:55. doi: 10.1186/s13104-020-4919-3, PMID: 32019577 PMC7001199

[ref14] Feinkohl I. Winterer G. Spies C. D. Pischon T. (2017). Cognitive reserve and the risk of postoperative cognitive dysfunction. Dtsch. Arztebl. Int. 114, 110–117. doi: 10.3238/arztebl.2017.0110, PMID: 28302254 PMC5359463

[ref15] Fischer X. MWT-A Testmappe. Balingen, Germany: Spitta GmbH (2001)

[ref16] Gaudreau J. D. Gagnon P. Harel F. Tremblay A. Roy M. A. (2005). Fast, systematic, and continuous delirium assessment in hospitalized patients: the nursing delirium screening scale. J. Pain Symptom Manag. 29, 368–375. doi: 10.1016/j.jpainsymman.2004.07.009, PMID: 15857740

[ref17] Gauggel S. Birkner B. (1999). Validität und Reliabilität einer deutschen version der Geriatrischen Depressionsskala (GDS) [validity and reliability of a German version of the geriatric depression scale (GDS)]. Z. Klin. Psychol. 28, 18–27. doi: 10.1026//0084-5345.28.1.18

[ref18] Hestermann U. Backenstrass M. Gekle I. Hack M. Mundt C. Oster P.. (2009). Validation of a German version of the confusion assessment method for delirium detection in a sample of acute geriatric patients with a high prevalence of dementia. Psychopathology 42, 270–276. doi: 10.1159/000224151, PMID: 19521144

[ref19] Hill A. K. Venegas J. Clark E. (2018). “Wechsler test of adult Reading” in Encyclopedia of clinical neuropsychology. eds. Kreutzer J. S. DeLuca J. Caplan B. (Cham: Springer), 3709–3711.

[ref20] Impact of High Cholesterol on Vascular Health. (n.d.) Surgical Care Affiliates (SCA Health). Available at: siouxlandvascular.com/impact-of-high-cholesterol-on-vascular-health (Accessed August 20, 2023).

[ref21] International Standard Classification of Education ISCED (1997). Available at: uis.unesco.org/sites/default/files/documents/international-standard-classification-of-education-isced-1997-en_0.pdf (Accessed August 20, 2023).

[ref22] International Standard Classification of Education ISCED (2011). Available at: uis.unesco.org/sites/default/files/documents/international-standard-classification-of-education-isced-2011-en.pdf (Accessed August 20, 2023).

[ref23] Kastaun S. Gerriets T. Schwarz N. P. Yeniguen M. Schoenburg M. Tanislav C.. (2016). The relevance of postoperative cognitive decline in daily living: results of a 1-year follow-up. J. Cardiothorac. Vasc. Anesth. 30, 297–303. doi: 10.1053/j.jvca.2015.12.008, PMID: 27013120

[ref24] Kitsis P. Zisimou T. Gkiatas I. Kostas-Agnantis I. Gelalis I. Korompilias A.. (2022). Postoperative delirium and postoperative cognitive dysfunction in patients with elective hip or knee arthroplasty: a narrative review of the literature. Life (Basel). 12:314. doi: 10.3390/life12020314 35207601 PMC8878498

[ref25] Ko K. Yi D. Byun M. S. Lee J. H. Jeon S. Y. Kim W. J.. (2022). Cognitive reserve proxies, Alzheimer pathologies, and cognition. Neurobiol. Aging 110, 88–95. doi: 10.1016/j.neurobiolaging.2021.10.005, PMID: 34879329 PMC9234822

[ref26] Kunicki Z. J. Ngo L. H. Marcantonio E. R. Tommet D. Feng Y. Fong T. G.. (2023). Six-year cognitive trajectory in older adults following major surgery and delirium. JAMA Intern. Med. 183, 442–450. doi: 10.1001/jamainternmed.2023.0144, PMID: 36939716 PMC10028541

[ref27] Lehrl S Merz J Bukhard G. Mehrfachwahl-Wortschatz-Intelligenztest MWT-A. Göttingen, Germany: Hogrefe (1991).

[ref28] Lehrl S. Triebig G. Fischer B. (1995). Multiple choice vocabulary test MWT as a valid and short test to estimate premorbid intelligence. Acta Neurol. Scand. 91, 335–345. doi: 10.1111/j.1600-0404.1995.tb07018.x 7639062

[ref29] Levin P. (2007). “Postoperative delirium” in Complications in anesthesia. ed. Atlee J. L.. 2nd ed (Berkley, CA/USA: Elsevier Academic Press), 888–889.

[ref30] Lütz A. Radtke F. M. Franck M. Seeling M. Gaudreau J. D. Kleinwächter R.. (2008). Die Nursing Delirium Screening Scale (Nu-DESC) - Richtlinienkonforme Ubersetzung für den deutschsprachigen Raum [The Nursing Delirium Screening Scale (NU-DESC)]. Anasthesiol. Intensivmed. Notfallmed. Schmerzther. 43, 98–102. doi: 10.1055/s-2008-1060551, PMID: 18293243

[ref31] Medi C. Evered L. Silbert B. Teh A. Halloran K. Morton J.. (2013). Subtle post-procedural cognitive dysfunction after atrial fibrillation ablation. J. Am. Coll. Cardiol. 62, 531–539. doi: 10.1016/j.jacc.2013.03.073 23684686

[ref32] MNA-International GroupKaiserM. J.BauerJ. M.RamschC.UterW.GuigozY.. (2009). Validation of the Mini nutritional assessment short-form (MNA-SF): a practical tool for identification of nutritional status. J. Nutr. Health Aging 13, 782–788. doi: 10.1007/s12603-009-0214-7, PMID: 19812868

[ref33] Moller J. T. Cluitmans P. Rasmussen L. S. Houx P. Rasmussen H. Canet J.. (1998). Long-term postoperative cognitive dysfunction in the elderly ISPOCD1 study. ISPOCD investigators. International study of post-operative cognitive dysfunction. Lancet 351, 857–861. doi: 10.1016/S0140-6736(97)07382-0, PMID: 9525362

[ref34] Montine T. J. Cholerton B. A. Corrada M. M. Edland S. D. Flanagan M. E. Hemmy L. S.. (2019). Concepts for brain aging: resistance, resilience, reserve, and compensation. Alzheimers Res. Ther. 11:22. doi: 10.1186/s13195-019-0479-y, PMID: 30857563 PMC6410486

[ref3] National Academy of Sciences, Committee on Occupational Classification and Analysis (1981) DICTIONARY OF OCCUPATIONAL TITLES (DOT): PART I - CURRENT POPULATION SURVEY, APRIL 1971, AUGMENTED WITH DOT CHARACTERISTICS, AND PART II - FOURTH EDITION DICTIONARY OF DOT SCORES FOR 1970 CENSUS CATEGORIES [Computer file]. Washington, DC: U.S. Dept. of Commerce, Bureau of the Census [producer], 197?. Ann Arbor, MI: Inter-university Consortium for Political and Social Research [distributor].

[ref35] Relander K. Hietanen M. Rantanen K. Rämö J. Vento A. Saastamoinen K. P.. (2020). Postoperative cognitive change after cardiac surgery predicts long-term cognitive outcome. Brain Behav. 10:e01750. doi: 10.1002/brb3.1750, PMID: 32681544 PMC7507551

[ref36] Rudolph J. L. Marcantonio E. R. (2011). Review articles: postoperative delirium: acute change with long-term implications. Anesth. Analg. 112, 1202–1211. doi: 10.1213/ANE.0b013e3182147f6d 21474660 PMC3090222

[ref37] Rundshagen I. (2014). Postoperative cognitive dysfunction. Dtsch. Arztebl. Int. 111, 119–125. doi: 10.3238/arztebl.2014.0119, PMID: 24622758 PMC3959222

[ref38] Saczynski J. S. Inouye S. K. Kosar C. Tommet D. Marcantonio E. R. Fong T.. (2014). Cognitive and brain reserve and the risk of postoperative delirium in older patients. Lancet Psychiatry 1, 437–443. doi: 10.1016/S2215-0366(14)00009-1, PMID: 25642414 PMC4307596

[ref39] Saudek C. D. Brick J. C. (2009). The clinical use of hemoglobin A1c. J. Diabetes Sci. Technol. 3, 629–634. doi: 10.1177/193229680900300402, PMID: 20144304 PMC2769940

[ref40] Sawamura S. (2017). “Diagnosis of POD and POCD” in Anesthesia and neurotoxicity. ed. Morimoto Y. (Tokyo, Japan: Springer), 105–120.

[ref41] Schmand B. Bakker D. Saan R. Louman J. (1991). De Nederlandse Leestest voor Volwassenen: een maat voor het premorbide intelligentieniveau [The Dutch Reading Test for Adults: a measure of premorbid intelligence level]. Tijdschr. Gerontol. Geriatr. 22, 15–19. PMID: 1877068

[ref42] Schmitt E. M. Saczynski J. S. Kosar C. M. Jones R. N. Alsop D. C. Fong T. G.. (2015). The successful aging after elective surgery (SAGES) study: cohort description and data quality procedures. J. Am. Geriatr. Soc. 63, 2463–2471. doi: 10.1111/jgs.13793, PMID: 26662213 PMC4688907

[ref43] Scott J. E. Mathias J. L. Kneebone A. C. Krishnan J. (2017). Postoperative cognitive dysfunction and its relationship to cognitive reserve in elderly total joint replacement patients. J. Clin. Exp. Neuropsychol. 39, 459–472. doi: 10.1080/13803395.2016.1233940 27676314

[ref44] Smith J. P. (2007). The impact of socioeconomic status on health over the life-course. J. Hum. Resour. XLII, 739–764. doi: 10.3368/jhr.XLII.4.739

[ref45] Statistisches Bundesamt, (2020) Operations in hospitals in 2019: 38% of patients with full in-patient treatment were operated. Available at: https://www.destatis.de/EN/Press/2020/11/PE20_437_231.html (Accessed August 20, 2023).

[ref46] Steinmetz J. Christensen K. B. Lund T. Lohse N. Rasmussen L. S. the ISPOCD Group (2009). Long-term consequences of postoperative cognitive dysfunction. Anesthesiology 110, 548–555. doi: 10.1097/ALN.0b013e318195b569 19225398

[ref47] Stern Y. Barnes C. A. Grady C. Jones R. N. Raz N. (2019). Brain reserve, cognitive reserve, compensation, and maintenance: operationalization, validity, and mechanisms of cognitive resilience. Neurobiol. Aging 83, 124–129. doi: 10.1016/j.neurobiolaging.2019.03.022, PMID: 31732015 PMC6859943

[ref48] Wang Y. Zhu H. Xu F. Ding Y. Zhao S. Chen X. (2023). The effect of anesthetic depth on postoperative delirium in older adults: a systematic review and meta-analysis. BMC Geriatr. 23:719. doi: 10.1186/s12877-023-04432-w, PMID: 37932677 PMC10629190

[ref49] Whitlock E. L. Vannucci A. Avidan M. S. (2011). Postoperative delirium. Minerva Anestesiol. 77, 448–456. PMID: 21483389 PMC3615670

[ref50] Winterer G. Androsova G. Bender O. Boraschi D. Borchers F. Dschietzig T. B.. (2018). Personalized risk prediction of postoperative cognitive impairment - rationale for the EU-funded BioCog project. Eur. Psychiatry 50, 34–39. doi: 10.1016/j.eurpsy.2017.10.004, PMID: 29398565

[ref51] Ying Tan A. M. Amokao D. (2013). Postoperative cognitive dysfunction after cardiac surgery. Contin. Educ. Anaesth. Crit. Care Pain 13, 218–223. doi: 10.1093/bjaceaccp/mkt022

[ref52] Zhang W. Sun Y. Liu Y. Qiu W. Ye X. Zhang G.. (2017). A nursing protocol targeting risk factors for reducing postoperative delirium in patients following coronary artery bypass grafting: results of a prospective before-after study. Int J Nurs Sci. 4, 81–87. doi: 10.1016/j.ijnss.2017.02.002, PMID: 31406724 PMC6626138

