# Supplementary material for: Cognitive reserve and the risk of postoperative neurocognitive disorders in older age
Source: Front Aging Neurosci. 2024 Feb 5;15:1327388. doi: 10.3389/fnagi.2023.1327388 (PMC10875020; doi:10.3389/fnagi.2023.1327388)
Supplement: Supplementary file 1 [file Data_Sheet_1.docx]

**Supplemental material for online only: Cognitive reserve and the risk of postoperative neurocognitive disorders in older age –**

# Assessment of Pre-morbid Intelligence Quotient

Vocabulary skills are robust to age related cognitive decline and therefore used for approximation of pre-morbid Intelligence Quotient (IQ). In the Mehrfachwortschatztest A (MWT-A) [1], patients were presented with 37 items in ascending difficulty level. Each item consisted of five words: one real target word and four nonsense distractors. For each item, the patient marked the target word, with one point given for each correctly identified word. There was no time limit on this task. In the Dutch Adult Reading Test (DART) [2], patients read 100 irregularly pronounced words aloud to a nurse or study physician. Each correctly pronounced word was rated one point. MWT-A scores ranged from 0–37 and DART scores from 0–100. DART and MWT-A scores were then converted to intelligence quotient (IQ) ranks based on published norms [2, 3].

# Assessment of Occupational Complexity

The Dictionary of Occupational Titles (DOT) is an online resource made available by the US Department of Labor [4]. Every job in this database has been assigned a unique nine-digit code (e.g., Nurse, general duty: 075.364-010), which defines each occupation in a systematic fashion, making it easy to compare different jobs. The first three digits of each code pertain to the occupational group in which the job is included. The first digit relates to the occupational category, the second to the specific “division”, and the third to a smaller occupational “group”. For example, 075, the first three digits in the code for “nurse, general duty”: the first digit, 0, indicates that the job is in the category of professional, technical, and managerial operations; the second digit, 7, indicates a job in the medical and health area, while the third digit, 5, denotes a registered nurse. The middle three digits of the code (digits 4–6) rate the “Worker Functions” of a job: every job requires workers to function in relation to data, people, and things to differing degrees. The degree of complexity with respect to data, people, and things of each job is rated in the DOT. Data complexity ratings range from 0–6, people complexity ratings from 0–8, and things complexity ratings from 0–7. Each patient’s last occupation was assigned three complexity ratings based on the ratings given in the DOT. Where no exact match was found, similar jobs were identified and used as a proxy. DOT ratings assign more complex jobs a lower numerical rating compared to less complex ones i.e., for each of data, people, and things complexity, “0” is the rating given to the most complex jobs in the DOT. The scales of IQ and International Standard Classification of Education (ISCED) (used to classify education level in this analysis) assign higher values to higher levels of IQ and education respectively. So, for the sake of consistency, DOT occupational complexity scores were reversed. A guide for interpreting these reversed occupational complexity scores is provided in Table S1.

Every effort was made to find the best possible match for all Dutch and German job titles in the analysis sample with the United States job titles in the DOT. Some occupa-tions had no close matches in the DOT and, though the nearest approximate match was identified and used as a proxy, this did not guarantee similarity in terms of data, people, and things complexity between the job in the analysis sample and the job in the DOT. Some last occupations given by patients e.g., student; housewife, were not listed in the DOT at all as these are not strictly classified as jobs. In order to not lose more patients from the analysis sample, the closest listed match was used e.g., research assistant and caregiver respectively.

A random subsample of 120 patient last occupations were rated by an independent coder, also using the DOT. A 2- way random model correlation showed high inter-rater reliability (correlation coefficient (ICC) for data complexity ratings 0.92, ICC for people complexity ratings 0.84, ICC for things complexity ratings 0.92). The value of ICC ranges between 0 and 1, where 1 indicates a perfect reliability between raters. Despite of the above described challenges the two raters had high levels of agreement.

# Assessment of Education level

Full patient educational background was self-reported and classified according to the International Standard Classification of Education (ISCED). At the Dutch study center, the ISCED 2011 classification system was used [5], though later converted to the ISCED 1997 [6] based on the ISCED 1997–2011 conversion table presented in Table S3. In Berlin, ISCED 1997 was used, which assigns categories ranging from 0 to 6 to each individual: 0, ‘pre-primary level of education’; 1, ‘primary’; 2, ‘lower secondary’; 3, ‘upper secondary’; 4, ‘post-secondary non-tertiary’; 5, ‘first stage tertiary’; 6, ‘second stage tertiary’. For the purpose of the current analysis, participants were grouped into ‘ISCED 1/2’, ‘ISCED 3/4’, and ‘ISCED 5/6’. None of the patients had ISCED 0.

# Assessment of postoperative delirium (POD)

# *Nu-DESC*

The Nu-DESC (Nursing Delirium Screening Checklist) was developed by Gaudreau et al [7] as a simple yet accurate delirium assessment instrument. The checklist covers five symptoms of delirium: disorientation, inappropriate behavior, inappropriate com- munication, illusions/hallucinations, and psychomotor retardation. Each of the symptoms is evaluated according to its presence and intensity and scored between zero and two points, for a maximum total of ten points. A total score of two or more points indicates that a patient is experiencing delirium. The Nu-DESC has been shown to have a sensitivity of 85.7% and specificity of 86.8% (Ibid.).

***CAM/CAM-ICU***

Originally developed by Inouye et al. [8], the Confusion Assessment Method (CAM) was designed to enable non-psychiatric clinicians to identify delirium in patients capable of verbal communication. It was later adapted to enable its use in the intensive care set- ting, where patients are often mechanically ventilated, rendering them nonverbal [9]. The contents and structure of the CAM is based on nine operationalized criteria from the 1987 Diagnostic and Statistical Manual of Mental Disorders (DSM-III-R): 1) acute onset, 2) inattention, 3) disorganized thinking, 4) altered level of consciousness, 5) disorientation,

1. memory impairment, 7) perceptual disturbances, 8) psychomotor agitation/retardation, and 9) altered sleep-wake cycle [8].

Trained raters perform a cognitive test and conduct a patient interview to score each

feature as present or absent. The presence of delirium is assessed via a diagnostic algorithm:

Of the four main features of delirium:

- 1. acute onset and fluctuating course, B) inattention, C) disorganized thinking, and D) altered level of consciousness – both criteria A and B as well as either or both of criteria C and D must be present.

A 2008 systematic review of current usage of the CAM screening showed an overall sensitivity of 94% and specificity of 89% [10].

The CAM-ICU screening process is shorter, taking around two minutes. A 2021 re- view of 29 studies using the CAM-ICU to detect ICU delirium found an overall sensitivity of 84% and specificity of 95% [11]. As ICU patients are often sedated, the first step of the CAM-ICU involves identifying the depth of sedation. This is assessed using another tool, the Richmond Agitation Sedation Scale (RASS). On this ten-level scale, zero (0) repre- sents a state of being calm and alert. A positive score indicates that a patient is agitated, spanning from restless (+1) to combative (+4). A negative score indicates a level of se- dation, spanning from drowsy (-1) to unarousable (-5). This score only becomes directly relevant to the delirium screening later but is carried out at this earlier point as the CAM- ICU screening requires patients to have a minimum level of consciousness: if the RASS score is equal to or greater than -3, the patient is considered conscious and the next stage of the CAM-ICU can be carried out. Patients with a RASS score of -4 or -5 are considered unconscious and should be assessed again at a later time. As patients in the ICU setting are nonverbal, the tests are structured around physical and yes-and-no responses that can be indicated by nodding or shaking the head. As in the CAM, the CAM-ICU screening is based on the four main features of delirium. The first feature, “acute onset or fluctuating course”, is assessed by means of observing the patient. The raters must ask themselves if the patient seems cognitively different than their baseline mental state, and if the patient’s mental state has fluctuated in the past 24 hours. Inattention, the second feature, is assessed using the Letters Attention Test. In this test, the rater reads the patient a set series of letters while holding the patient’s hand, instructing the patient to squeeze the tester’s hand each time the letter ‘A’ is read. The series varies according to language: SAVEAHAART in the Dutch version of the CAM-ICU [12] and ANANASBAUM in the German version [13]. The rater must count the number of errors made by the patient, which occur both when the patient squeezes the rater’s hand on a letter other than ‘A’ and when the patient fails to squeeze the rater’s hand on the letter ‘A’. The assessment of an “altered level of con- sciousness” refers back to the patient’s RASS score. At this point, if the rater answered ‘yes’ to either of the questions about the first feature, the patient made two or more errors on the Letters Attention Test, and their RASS score is anything other than zero, the patient is considered to be delirious, because, as we saw in the CAM, the first two criteria and either one of the latter two criteria is sufficient for a diagnosis of delirium. If features A (acute onset or fluctuating course) and B (inattention) are present but the patient’s RASS score is zero, the rater should move on to test the final feature: disorganised thinking. This is done by first asking four yes-or-no questions (e.g., “Will a stone float on water?”, “Are there fish in the sea?”, “Does one pound weigh more than two pounds?”, “Can you use a hammer to pound a nail?”). When a patient answers a question incorrectly, this is counted as an error. Then, the rater gives the patient several instructions (e.g., “Hold up this many fingers” while holding up two fingers. “Now do the same with the other hand” while not showing or “Add one more finger” if the patient is unable to move both arms), which the patient must follow. Failure to follow the entirety of the instructions also counts as an error. If the combined number of errors in this part of the test is greater than one, the patient is exhibiting disordered thinking and, in combination with features A and B, the CAM-ICU is positive for delirium [14].

***Chart review***

Patient charts were screened for entries made by nurses and medical doctors using key terms describing delirium, e.g. confused, agitated, drowsy, disoriented, delirious etc., and prescription of drugs for delirious symptom control.

# Assessment of Postoperative Cognitive Dysfunction (POCD)

The screen-based CANTAB assessment consisted of four tests: 1. Simple Reaction Time (SRT): A square is presented onscreen. The patient must respond to this stimulus by pressing a button as fast as possible. 2. Verbal Recall Memory (VRM): A list of 12 words is shown one by one. The patient is immediately asked to freely recall as many of the words shown as possible. 20 minutes after the initial presentation of the words, the patient is asked to identify the words they have seen previously, among a 24-word list containing 12 true words and 12 distractors. 3. Spatial Span (SSP): White squares presented onscreen change color in sequence. At the end of a sequence, the patient must select the boxes in the order that they changed color. 4. Paired Associate Learning (PAL): Boxes displayed onscreen open one at a time in a randomized order to reveal one or more patterns “inside” the boxes. The patient must then match the pattern(s) shown in the middle of the screen to the box where the pattern was initially shown. The two non-screen-based tests involved: 1. Trail-Making-Test: Using a pencil and paper, the patient must connect a sequence of consecutive targets together. In the first part of the test (TMT-A), targets are numbered (1, 2, 3, . . . ) and must be connected in numerical order. In the second part of the test (TMT-B), numbers and letters must be connected in an alternating fashion (1, A, 2, B, 3, C, . . . ). 2. Grooved Pegboard Test (GPT): Using their dominant hand, the patient is asked to insert 25 pegs into holes in a board as quickly as possible (GPT is also performed with the non-dominant hand, but values performance with the dominant hand only were used for evaluation). The pegs have a key along one side of them, so they must be rotated to match each of the randomly aligned holes in the board. From these tests, the following cognitive test parameters were used to assess cognitive function. The cognitive function tested by each parameter is given in brackets: 1. Mean correct latency in the SRT (processing speed) 2. Number of words correctly remembered in the VRM (free recall) 3. Number of words correctly identified after delay in the VRM (verbal memory/late recall) 4. Span length in the SSP (working memory) 5. First trial memory score in the PAL (visual memory) 6. Completion time for the TMT-B (executive function) 7. Completion time for the GPT (fine motor skills) The test parameters from the SRT, GPT, and TMT-B tests were log-transformed and sign-reversed before calculation to achieve an approximate normal distribution and a correspondence of higher scores with better cognitive performance. As recommended in the test manual, GPT completion times of more than 300 seconds were removed during plausibility checks. POCD was defined according to the Reliable Change Index (RCI), which shows a patient’s performance change in a cognitive test after surgery, compared to their performance in the same test at baseline i.e., in relation to the mean change in test performance in a non-operated comparison group. Following Rasmussen et al. 2001 (Table 3) [15], the POCD criteria were considered fulfilled for the purpose of the BioCog study if a patient’s performance deviated by more than -1.96 in the RCI of a single test parameter or the compound RCI of all test parameters. This approach treats POCD as a dichotomous variable the intention being to use this classification as a diagnosis in the clinical context. Using the RCI enabled adjusting for natural variability in cognitive performance and learning effects during repeated cognitive testing. The RCI was calculated for each test parameter as follows: *RCI*= ∆*X−*∆*XcSD*(∆*Xc*) where ∆X is the difference in post-surgery scores compared to the baseline and the mean test score difference between the corresponding time points in the non-operated comparison group. RCI is to the standard deviation (SD) of mean differences in the non-operated comparison group SD (∆*Xc*). The compound RCI for each patient was defined as the sum of all in relation to the SD of the sum of all in the non-operated comparison group: *compoundRCI*= Σ(*RCI*)*SD*(Σ(*RCIc*)). The cognitive tests chosen had a moderate-to-good retest-reliability in the non-operated comparison group (ICC between baseline and 3 months *≥* 0.75 based on a mean of multiple measurements, absolute-agreement, 2-way mixed-effects models): VRM free recall (ICC 0.68 [confidence interval (CI) 0.43, 0.82]), VRM delayed recognition (ICC 0.85 [CI 0.72, 0.92]), PAL (ICC 0.78 [CI 0.61,

0.88]), TMT-B (ICC 0.78 [CI 0.59, 0.88]), and GPT (ICC 0.92 [CI 0.86, 0.96]). Detailed information on the stability of neuropsychologial test performance of our non-operated comparison group can be found in a publication of our working group [16]. The applied research algorithm for dichotomization of cognitive data is accessible as R Package on- line [17].

TABLE S1

Description of occupational complexity levels with data, people, and things

| **Data** | **People** | **Things** |
| --- | --- | --- |
| 0 Comparing | 0 Taking instructions/helping | 0 Handling |
| 1 Copying | 1 Serving | 1 Feed-offbearing |
| 2 Computing | 2 Speaking-signalling | 2 Tending |
| 3 Compiling | 3 Persuading | 3 Manipulating |
| 4 Analysing | 4 Diverting | 4 Driving-operating |
| 5 Coordinating | 5 Supervising | 5 Operating-controlling |
| 6 Synthesizing | 6 Instructing | 6 Precision working |
|  | 7 Negotiating | 7 Setting up |
|  | 8 Mentoring |  |

TABLE S2

CR variables of Male and Female subjects

|  | **Male (n=408)** | **Female (n=305)** | **F-value** | **p-value** |
| --- | --- | --- | --- | --- |
| **IQ**  **[Mean ± SD]** | 111.7 ± 14.4 | 111.3 ± 14.9 | 0.14 | .713 |
|  |  |  |  |  |
| **Occupational complexity**  **[Median (IQR)]** |  |  | **Χ²**  **(1, n=713)** |  |
| **Data** | 5 (3-5) | 3 (3-5) | 37.73 | <.001 |
| **People** | 2 (2-5) | 2 (2-3) | 5.58 | .002 |
| **Things** | 3 (0-6) | 3 (0-5) | 0.93 | .336 |
|  |  |  |  |  |
| **Education level**  **[Absolute number (Relative frequency)]** |  |  | **Χ²**  **(2, n=713)** | **p-value** |
| **ISCED 1/2** | 51 (12.5) | 58 (19.0) | 38.79 | ≤.001 |
| **ISCED 3/4** | 140 (34.3) | 156 (51.1) |  |  |
| **ISCED 5/6** | 217 (53.2) | 91 (29.8) |  |  |

Analyses of variance (ANOVAs) were used for comparison of pre-morbid IQ between male
and female patients (F- and p-values provided). Kruskall-Wallis-Tests were used for
comparison of occupational complexity variables between male and female
 patients (χ² and p-value provided). A χ²-test was used to examine the association
of education with sex (χ² and p-value provided).

TABLE S3

Correspondence between ISCED 2011 and ISCED 1997 levels

| **ISCED 2011** | **ISCED 1997** |
| --- | --- |
| ISCED 01 | - |
| ISCED 02 | ISCED 0 |
| ISCED level 1 | ISCED level 1 |
| ISCED level 2 | ISCED level 2 |
| ISCED level 3 | ISCED level 3 |
| ISCED level 4 | ISCED level 4 |
| ISCED level 5 | ISCED level 5 |
| ISCED level 6 |  |
| ISCED level 7 |  |
| ISCED level 8 | ISCED level 6 |
| *content of category has been slightly modified | |

ISCED: International Standard Classification of Education, sources are
accessible online [18, 19]

TABLE S4

Subject characteristics of the non-operated comparison group

| Total sample n = 114 | | | Median | IQR |
| --- | --- | --- | --- | --- |
| Age (years) | | | 71.0 | 67–76 |
| MMSE score (points) | | | 29.0 | 28–30 |
| GDS score (points) | | | 1.0 | 0–2 |
| BMI (kg/m2) | | | 26.14 | 24.27–29.3 |
| IQ | | | 111 | 104–123 |
| Occupational complexity | | data | 4 | 3-5 |
|  |  | people | 2 | 2-5 |
|  |  | things | 0 | 0-5 |
|  |  | | Absolute number | Relative frequency (%) |
| Female | | | 56 | 49 |
| Education level | ISCED 1/2 | | 33 | 29.7 |
|  | ISCED 3/4 | | 34 | 30.6 |
|  | ISCED 5/6 | | 44 | 39.6 |

MMSE: Mini-Mental-State-Exam; GDS: Geriatric Depression Scale;
BMI: Body Mass Index; IQ: Intelligence quotient;
ISCED: International Standard Classification of Education; IQ and
Education level data was missing for 3 subjects and not imputed.

TABLE S5

CR markers of subjects with peripheral vs. intraabdominal/-thoracic/-pelvic site of surgery

|  | **Peripheral (n=390)** | **Intra- abdominal/-thoracic/-pelvic (n = 323)** | **F-value** | **p-value** |
| --- | --- | --- | --- | --- |
| **IQ**  **[Mean ± SD]** | 111.9 ± 14.7 | 111.0 ± 14.5 | 0.702 | .403 |
|  |  |  |  |  |
| **Occupational complexity**  **[Median (IQR)]** |  |  | **Χ²**  **(1, n=713)** |  |
| **Data** | 2 (1-3) | 2 (1-3) | 0.04 | .848 |
| **People** | 6 (5-6) | 6 (3-6) | 0.50 | .478 |
| **Things** | 4 (1-7) | 7 (2-7) | 1.82 | .177 |
|  |  |  |  |  |
| **Education level**  **[Absolute number (Relative frequency)]** |  |  | **Χ²**  **(2, n=713)** | **p-value** |
| **ISCED 1/2** | 48 (14.9) | 61 (15.6) | 0.46 | 0.794 |
| **ISCED 3/4** | 131 (40.6) | 165 (42.3) |  |  |
| **ISCED 5/6** | 144 (44.6) | 164 (42.1) |  |  |

Analyses of variance (ANOVAs) were used for comparison of pre-morbid IQ between patients with peripheral vs. intraabdominal/-throacic/-pelvic site of surgery (F- and p-values provided). Kruskall-Wallis-Tests were used for
comparison of occupational complexity variables between patients with peripheral vs. intraabdominal/-throacic/-pelvic site of surgery (χ² and p-value provided). A χ²-test was used to examine the association
of education with site of surgery (χ² and p-value provided).


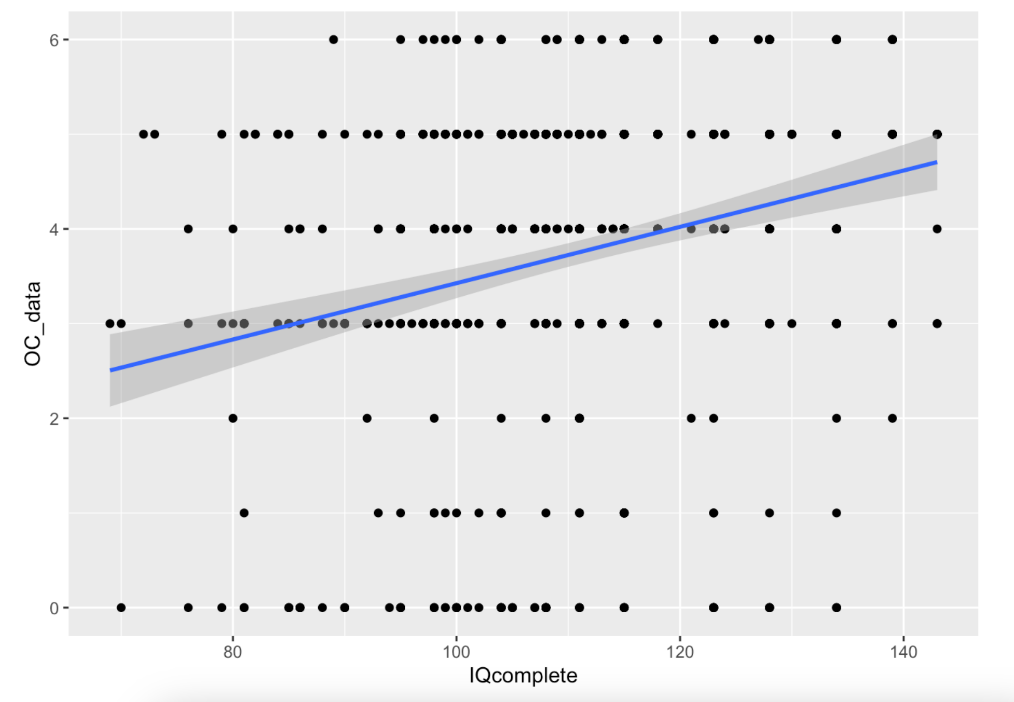


FIGURE S1: Scatterplot - correlation of occupational complexity “data” and IQ

**
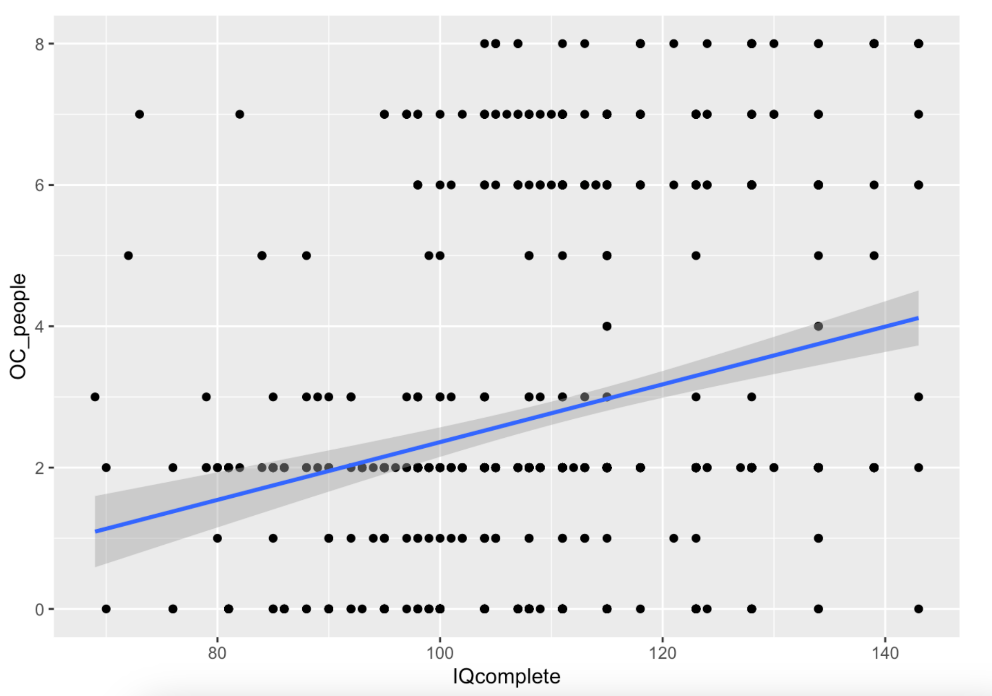
**

FIGURE S2: Scatterplot - correlation of occupational complexity “people” and IQ

**
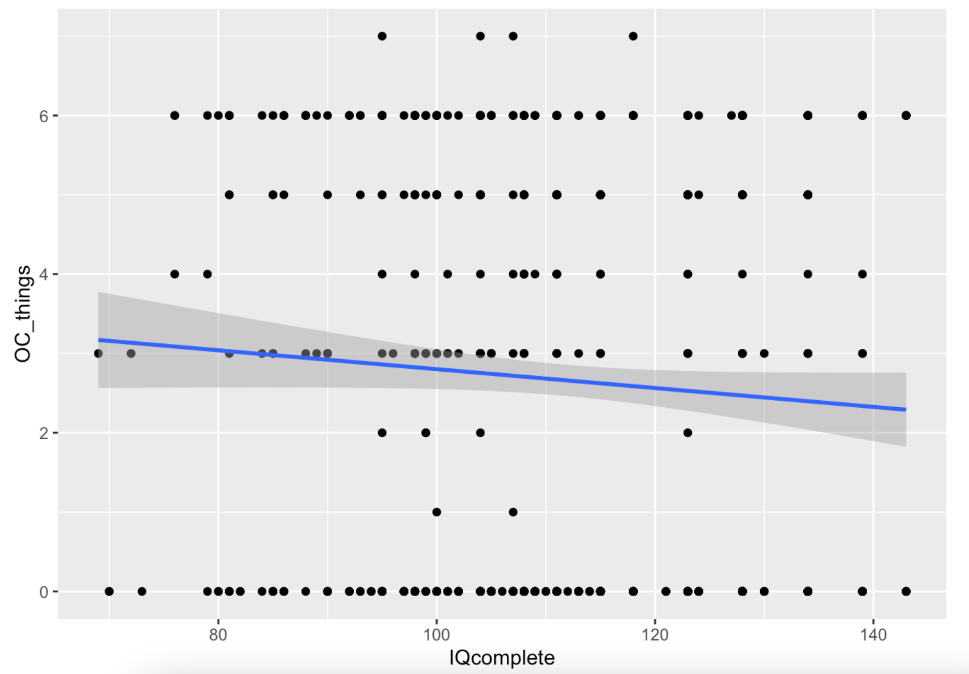
**

FIGURE S3: Scatterplot - correlation of occupational complexity “things” and IQ

# Association of Cognitive Reserve markers with mortality

Mortality was low (n=24/713; 3.4%), yet patients with a lower CR may have had increased mortality risk during the follow-up period. We therefore calculated the associations of CR markers with mortality, applying the Pearson χ² test for categorical variables and binary regression for IQ as continuous variable.

None of the CR markers was associated with mortality: Pre-morbid IQ (OR 0.992, CI 0.965-1.020; p=0.562); Occupational Complexity for “data” [χ² (6=7.810, p=0.252)], “people” [χ² (8=10.247, p=0.248)], “things” [χ² (7=5.286, p=0.625)]; education level (ISCED) [χ² (2=1.930, p=0.381)].

**References**

1. Lehrl S, Merz J, Bukhard G. Mehrfachwahl-Wortschatz-Intelligenztest MWT-A. Göttingen, Germany: Hogrefe 1991.
2. Schmand B, Bakker D, Saan R, Louman J. De Nederlandse Leestest voor Volwassenen: een maat voor het premorbide intelligentieniveau [The Dutch Reading Test for Adults: a measure of premorbid intelligence level]. Tijdschr Gerontol Geriatr. 1991 Feb;22(1):15-9.
3. Fischer X. MWT-A Testmappe. Balingen, Germany: Spitta GmbH 2001.
4. Dictionary of Occupational Titles [On-line]. [www.occupationalinfo.org](http://www.occupationalinfo.org). Accessed August 20, 2023.
5. International Standard Classification of Education ISCED 2011[On-line]. uis.unesco.org/sites/default/files/documents/international-standard-classification-of-education-isced-2011-en.pdf. Accessed August 20, 2023.
6. International Standard Classification of Education ISCED 1997 [On-line]. uis.unesco.org/sites/default/files/documents/international-standard-classification-of-education-isced-1997-en_0.pdf. Accessed August 20, 2023.
7. Gaudreau JD, Gagnon P, Harel F et al. Fast, systematic, and continuous delirium assessment in hospitalized patients: the nursing delirium screening scale. J Pain Symptom Manage. 2005 Apr;29(4):368-75.
8. Inouye SK, van Dyck CH, Alessi CA et al. Clarifying confusion: the confusion assessment method. A new method for detection of delirium. Ann Intern Med. 1990 Dec 15;113(12):941-8.
9. Ely EW, Margolin R, Francis J et al. Evaluation of delirium in critically ill patients: validation of the Confusion Assessment Method for the Intensive Care Unit (CAM-ICU). Crit Care Med. 2001 Jul;29(7):1370-9.
10. Wei LA, Fearing MA, Sternberg EJ et al. The Confusion Assessment Method: a systematic review of current usage. J Am Geriatr Soc. 2008 May;56(5):823-30.
11. Chen TJ, Chung YW, Chang HR et al. Diagnostic accuracy of the CAM-ICU and ICDSC in detecting intensive care unit delirium: A bivariate meta-analysis. Int J Nurs Stud. 2021 Jan;113:103782.
12. Vreeswijk R, Toornvliet AC, Bakker K et al. Validation of the dutch version of the Confusion Assessment Method (CAM-ICU) for delirium screening in the Intensive Care Unit. Netherlands Journal of Critical Care 2009; pp. 73–78.
13. Delir. Prävention – Früherkennung – Behandlung. Ein Ratgeber für Ärzte, Pflegepersonal, Patientenkoordinatoren und Einzelbetreuende [On-line]. viv[antes.de/](http://www.vivantes.de/) fileadmin/Pflege/PDFs/delir-ratgeber-krankenhaus-berlin_190515.pdf. Accessed August 22, 2023.
14. Confusion Assessment Method for the ICU (CAM-ICU): The Complete Training Manual [On-line]. www.icudelirium.org/resource-downloads/cam-icu-training-manual. Accessed August 22, 2023.
15. Rasmussen LS, Larsen K, Houx P et al. The International Study of Postoperative Cognitive Dysfunction. The assessment of postoperative cognitive function. Acta Anaesthesiol Scand. 2001 Mar;45(3):275-89.
16. Feinkohl I, Borchers F, Burkhardt S et al. Stability of neuropsychological test performance in older adults serving as normative controls for a study on postoperative cognitive dysfunction. BMC Res Notes. 2020 Feb 4;13(1):55.
17. Wiebach, J. (2021), R Package ’POCDr’. URL https://github.com/Wiebachj/ POCDr.
18. (2006), International Standard Classification of Education 1997. URL <http://uis.unesco.org/sites/default/files/documents/international-standard-> classification-of-education-1997-en_0.pdf.
19. (2012), International Standard Classification of Education 2011. URL <http://uis.unesco.org/sites/default/files/documents/international-standard-> classification-of-education-isced-2011-en.pdf.
